# Supplementary material for: Pyrimidine Schiff Bases: Synthesis, Structural Characterization and Recent Studies on Biological Activities
Source: Int J Mol Sci. 2024 Feb 8;25(4):2076. doi: 10.3390/ijms25042076 (PMC10889512; doi:10.3390/ijms25042076)
Supplement: Supplementary file 1 [file ijms-25-02076-s001.zip › ijms-2825578-supplementary.pdf]

Supplementary Materials

Pyrimidine Schiff Bases: Synthesis, Structural Characterization and Recent Studies on Biological Activities

Iwona Bryndal [1,*], Marcin Stolarczyk [1], Aleksandra Mikołajczyk [2], Magdalena Krupińska [2], Anna Pyra [3], Marcin Mączyński [1] and Agnieszka Matera-Witkiewicz [2]

[1] Department of Organic Chemistry and Drug Technology, Faculty of Pharmacy, Wroclaw Medical University, 211A Borowska, 50-556 Wrocław, Poland; marcin.stolarczyk@umw.edu.pl (M.S.); marcin.maczynski@umw.edu.pl (M.M.)

[2] Screening Biological Activity Assays and Collection of Biological Material Laboratory, Wroclaw Medical University, 211A Borowska, 50-556 Wrocław, Poland; aleksandra.mikolajczyk@umw.edu.pl (A.M.); magdalena.krupinska@umw.edu.pl (M.K.); agnieszka.matera-witkiewicz@umw.edu.pl (A.M.-W.)

[3] Faculty of Chemistry, University of Wroclaw, 14 Joliot-Curie, 50-383 Wrocław, Poland; anna.pyra@uwr.edu.pl

* Correspondence: iwona.bryndal@umw.edu.pl; Tel.: +48-71-784-02-36

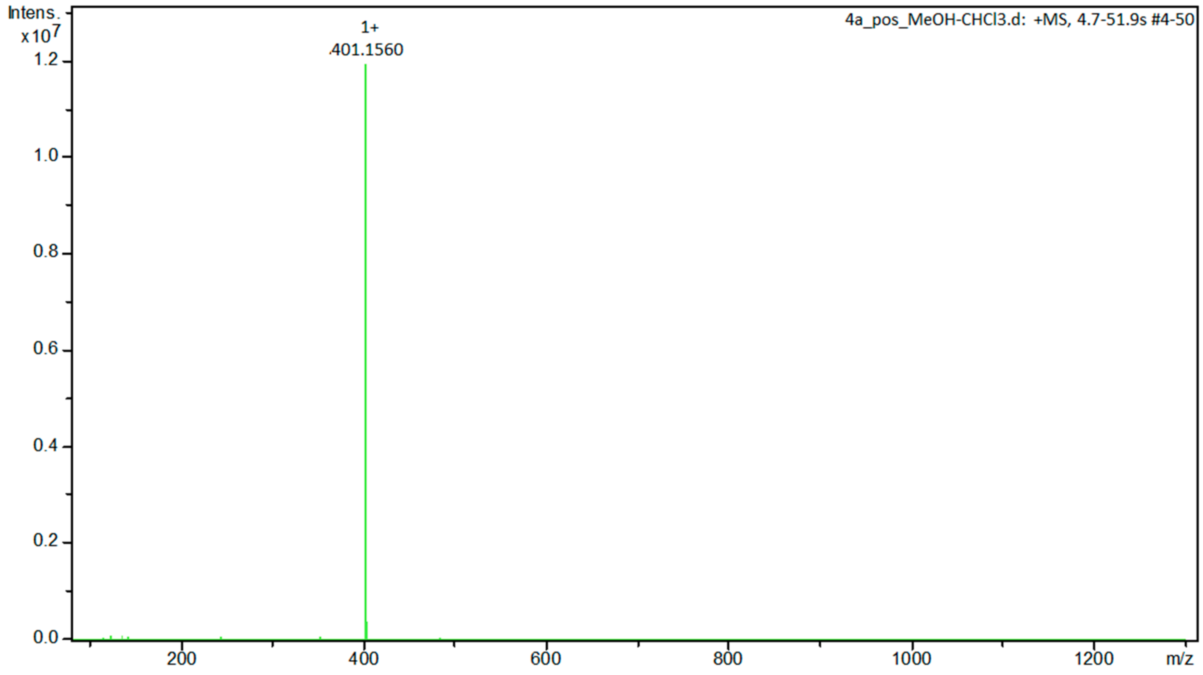

Figure S1. HR-ESI-MS spectra of compound **4a**.

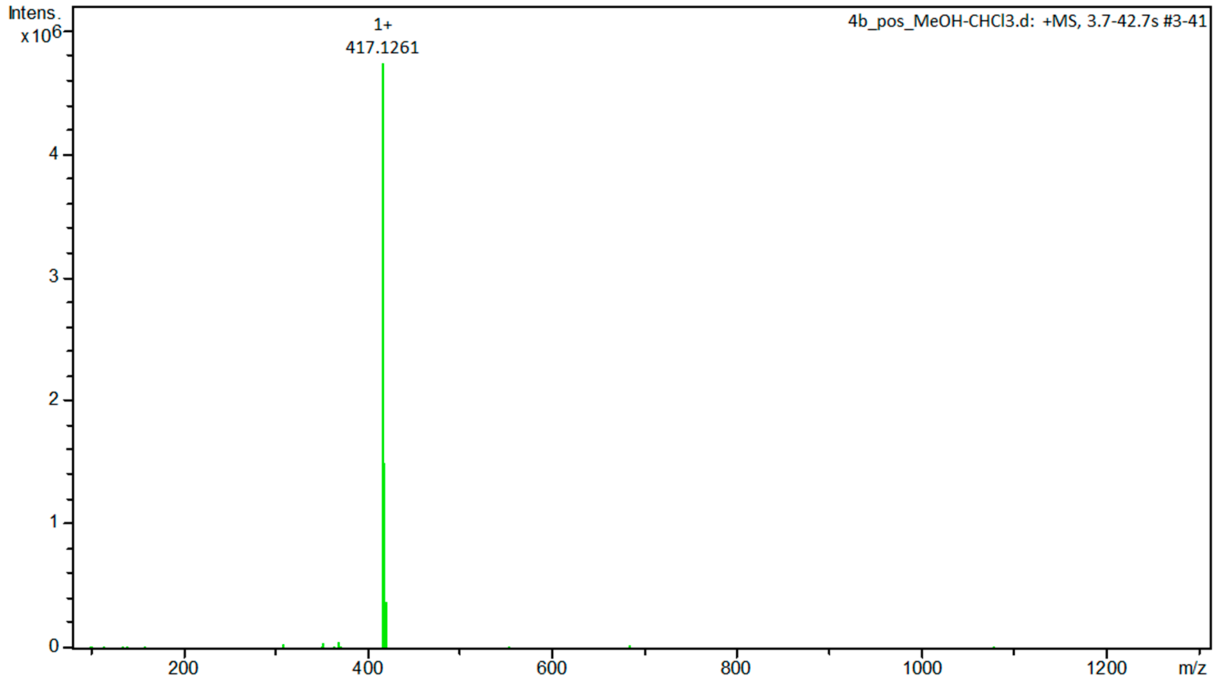

Figure S2. HR-ESI-MS spectra of compound **4b**.

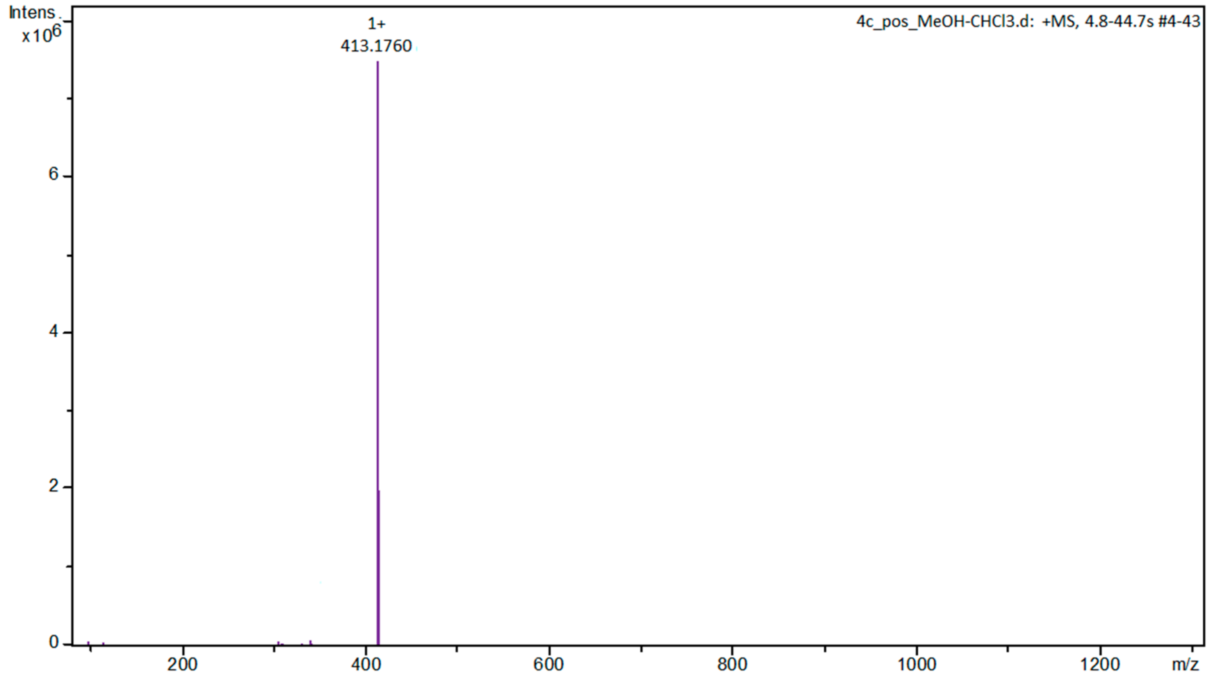

Figure S3. HR-ESI-MS spectra of compound **4c**.

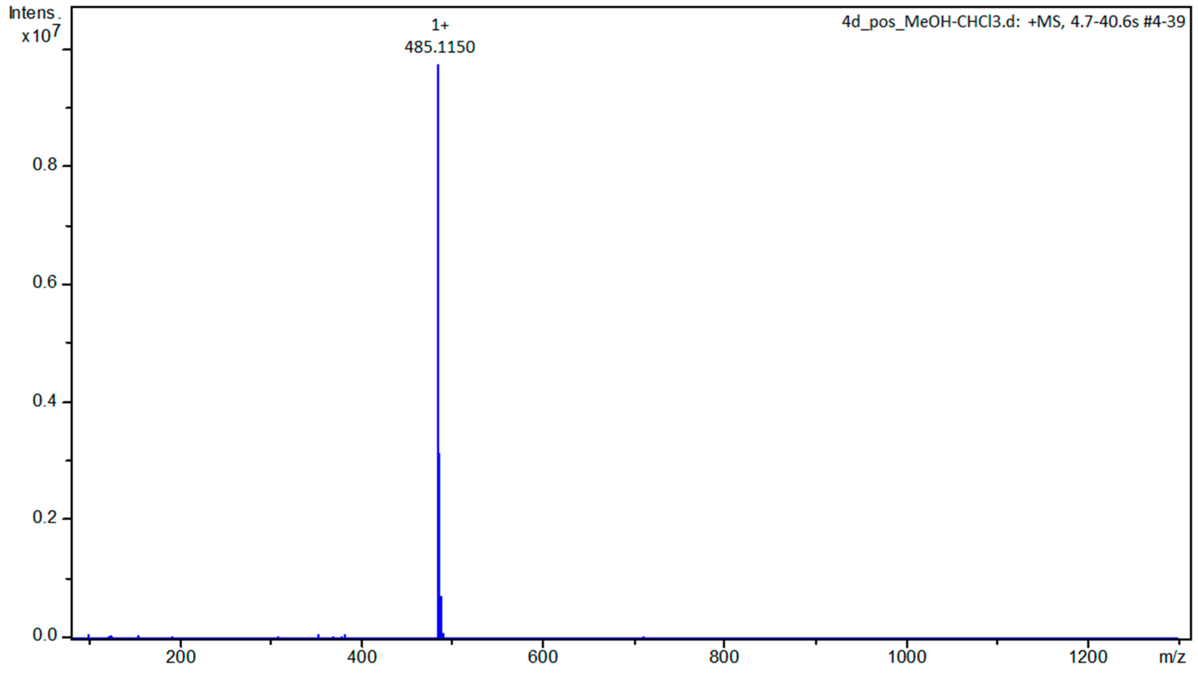

Figure S4. HR-ESI-MS spectra of compound **4d**.

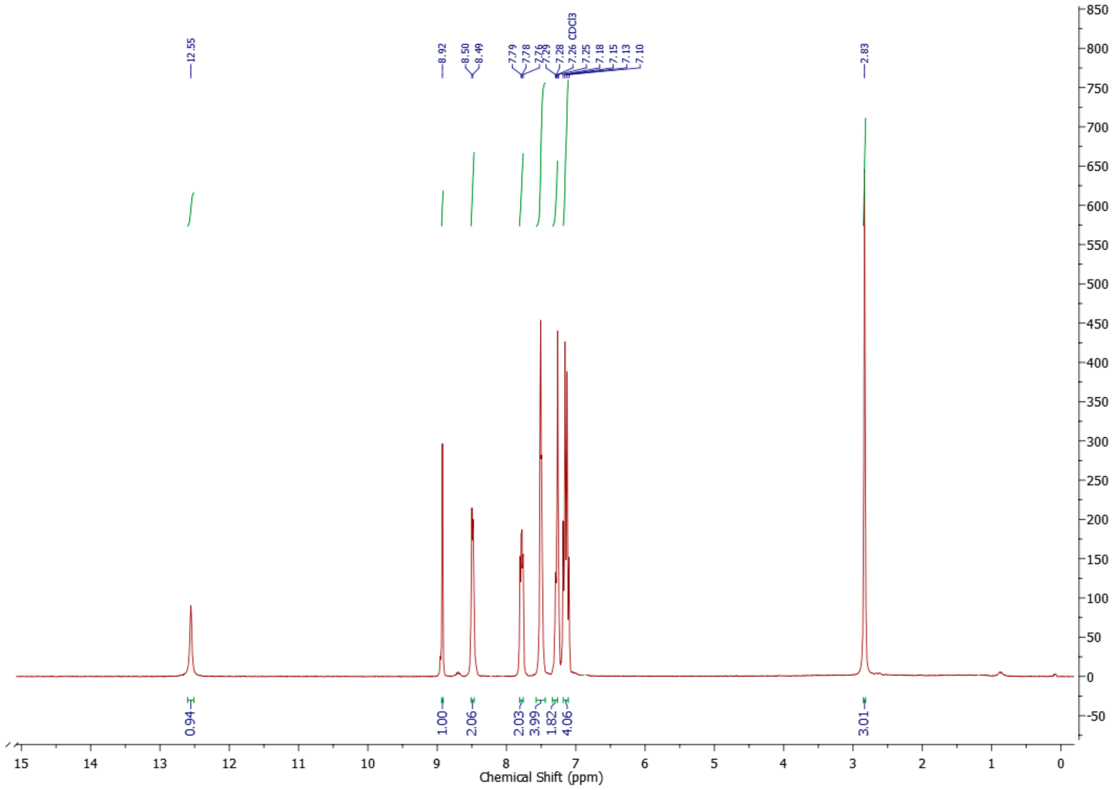

Figure S5. ^{1}H NMR spectrum of compound **4a** (300M Hz, $CDCl_3$).

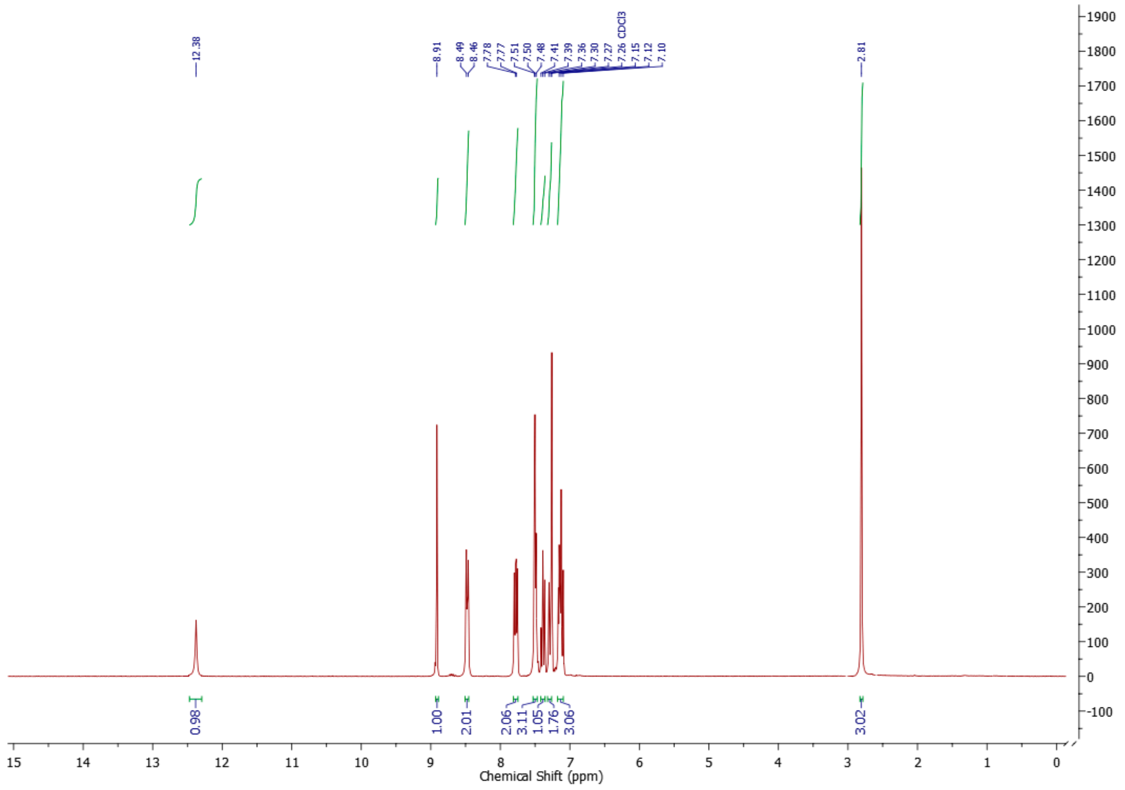

Figure S6. ^{1}H NMR spectrum of compound **4b** (300M Hz, $CDCl_3$).

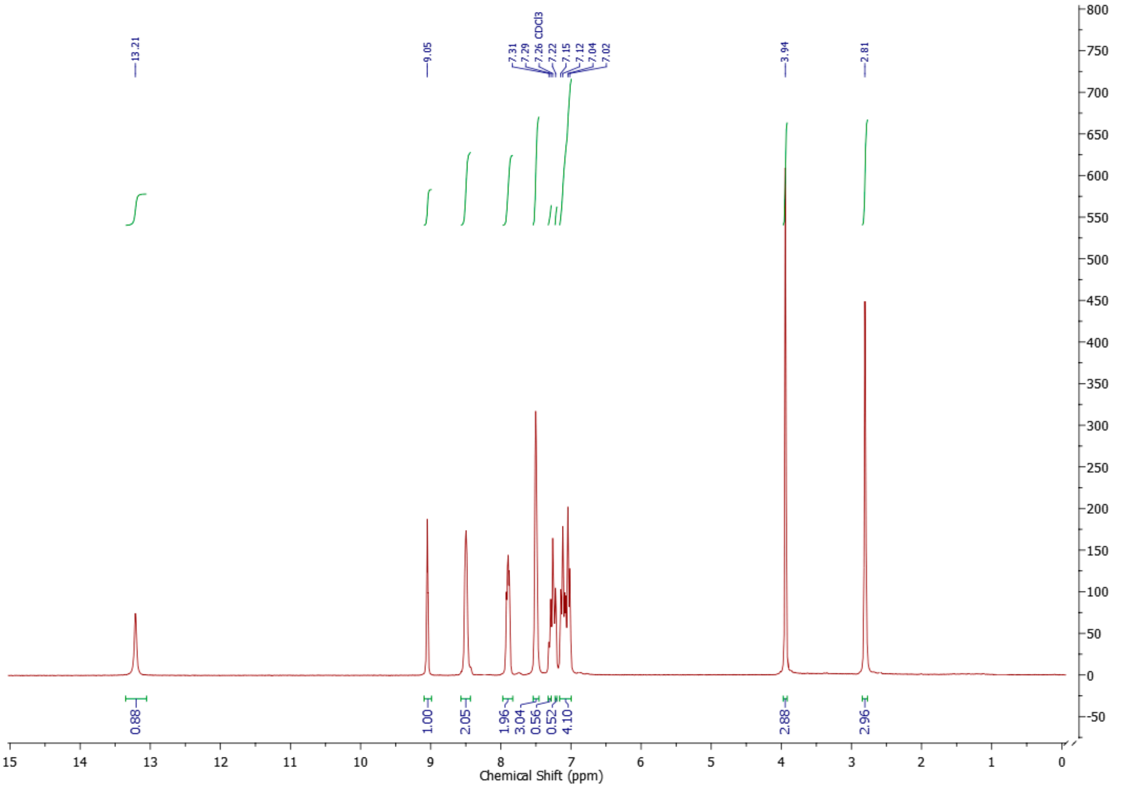

Figure S7. ^{1}H NMR spectrum of compound **4c** (300M Hz, $CDCl_3$).

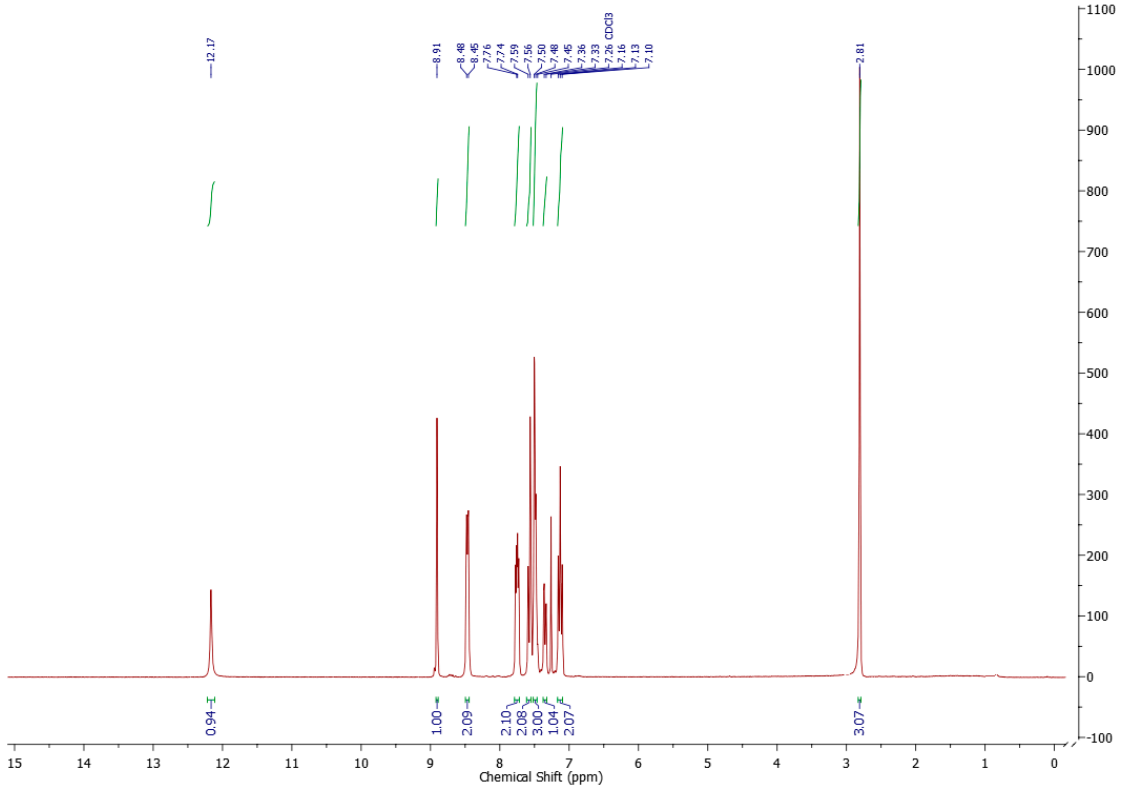

Figure S8. ^{1}H NMR spectrum of compound **4d** (300 MHz, $CDCl_3$).

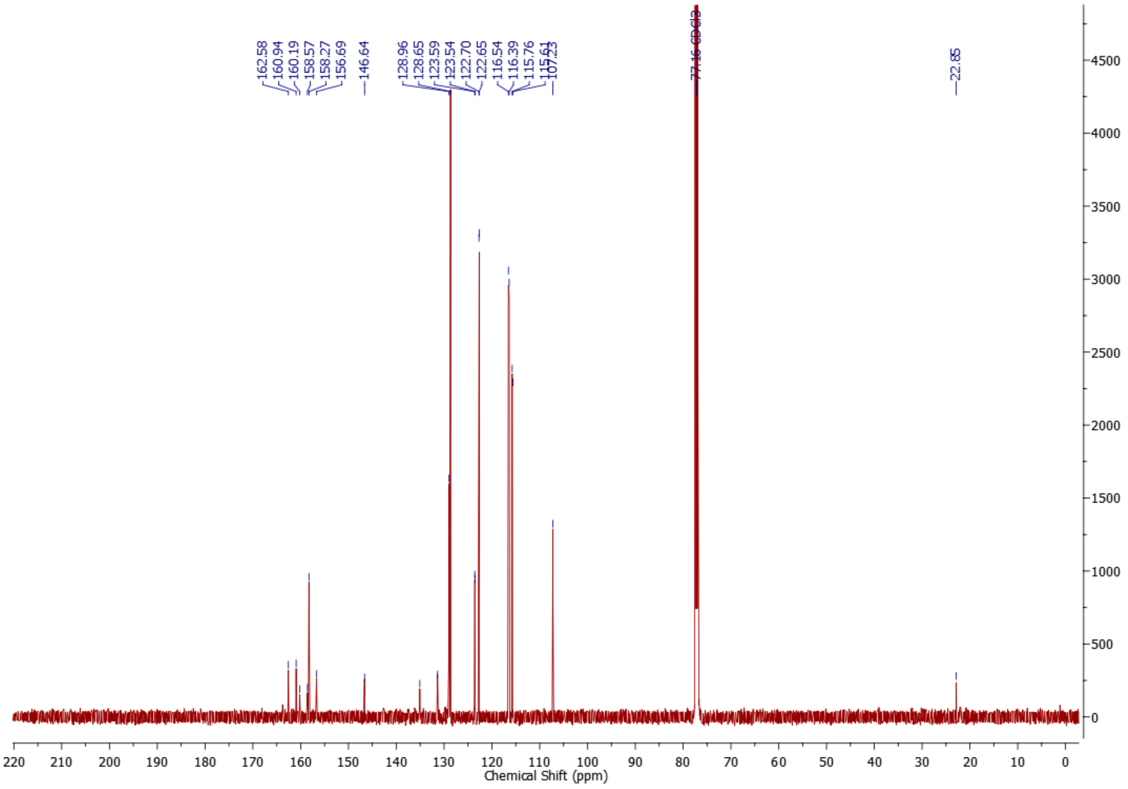

Figure S9. ^{13}C NMR spectrum of compound **4a** (150 MHz, $CDCl_3$).

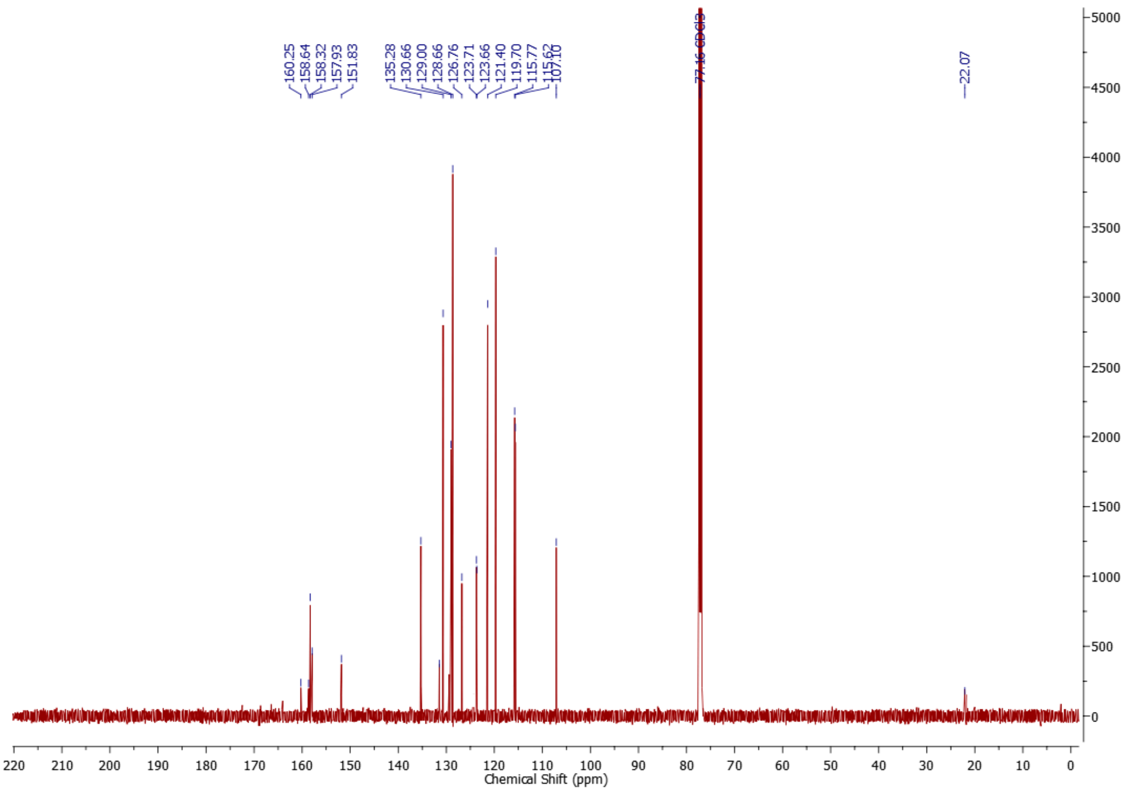

Figure S10. ^{13}C NMR spectrum of compound **4b** (150 MHz, $CDCl_3$).

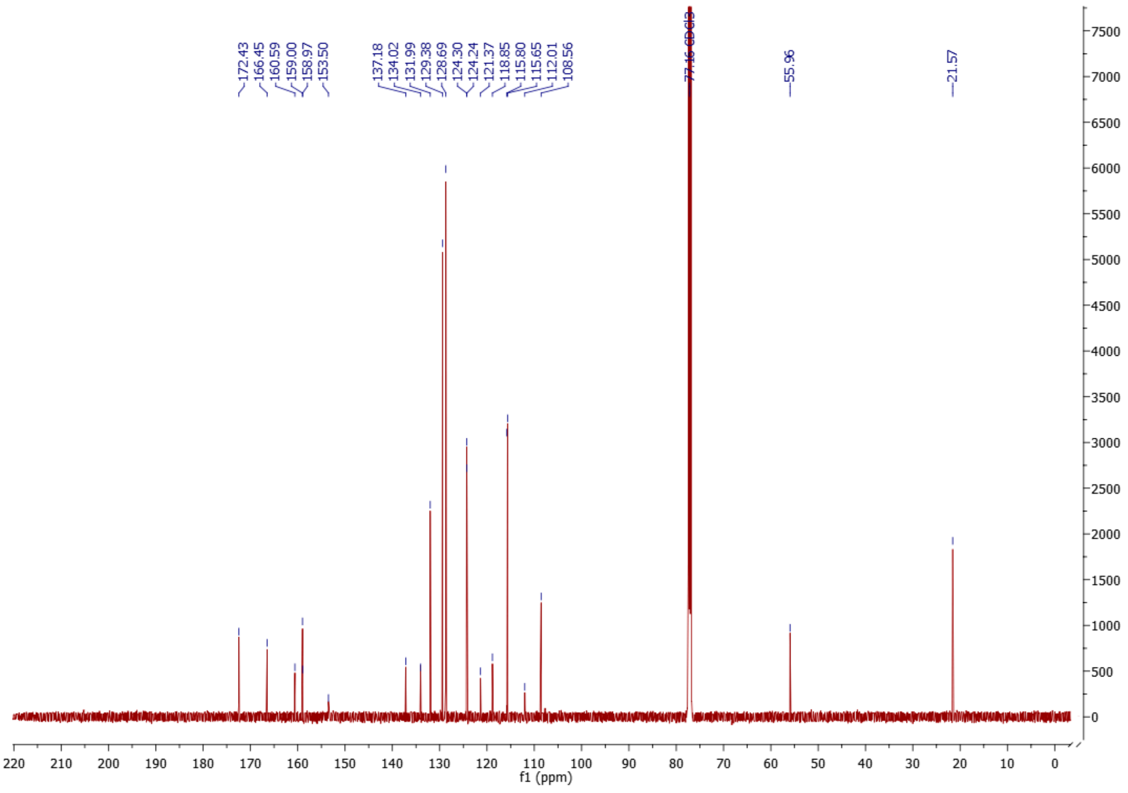

Figure S11. ^{13}C NMR spectrum of compound **4c** (150 MHz, $CDCl_3$).

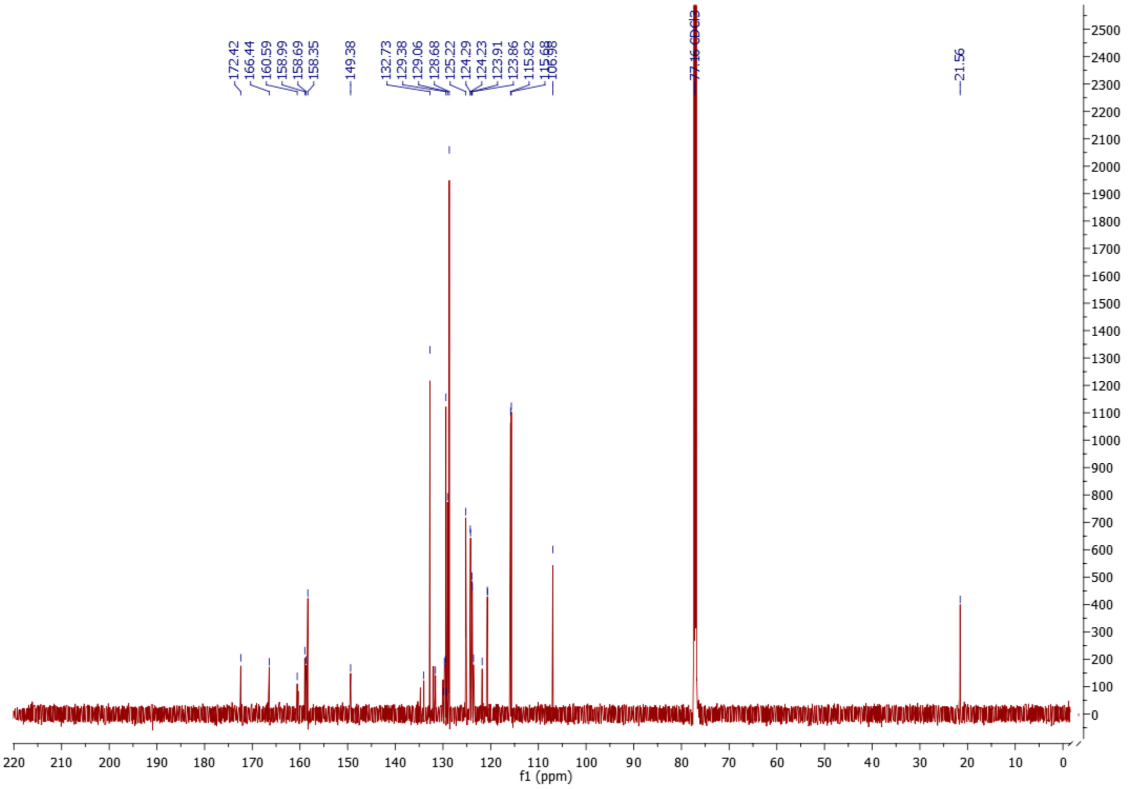

Figure S12. ^{13}C NMR spectrum of compound **4d** (150 MHz, $CDCl_3$).

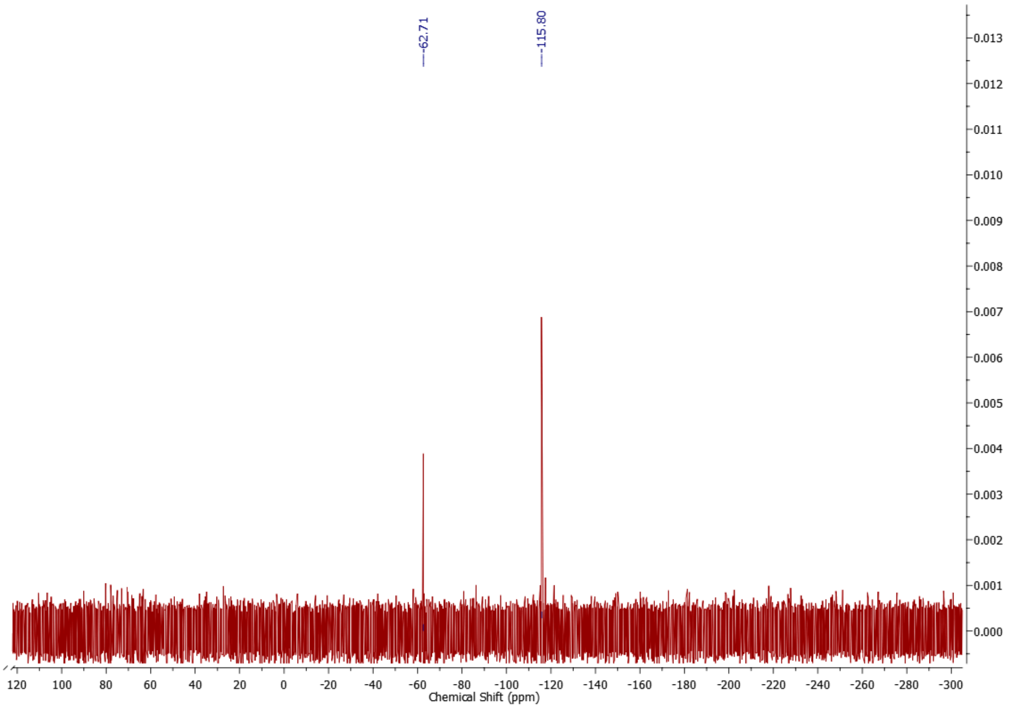

Figure S13. ^{19}F NMR spectrum of compound **4a** (471 MHz).

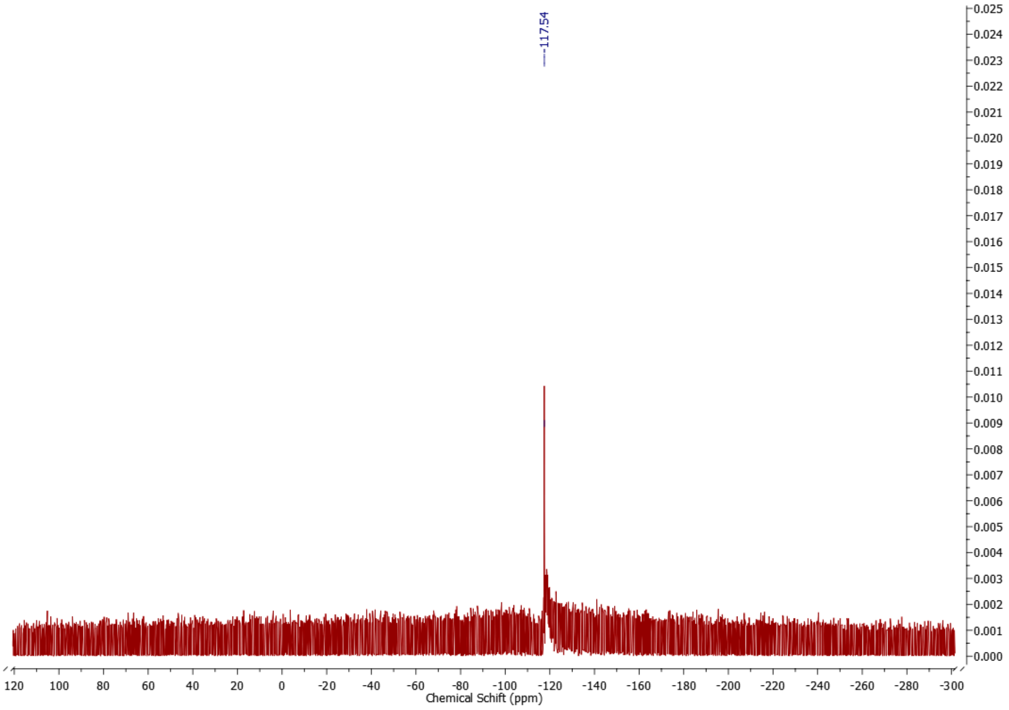

Figure S14. ^{19}F NMR spectrum of compound **4b** (471 MHz).

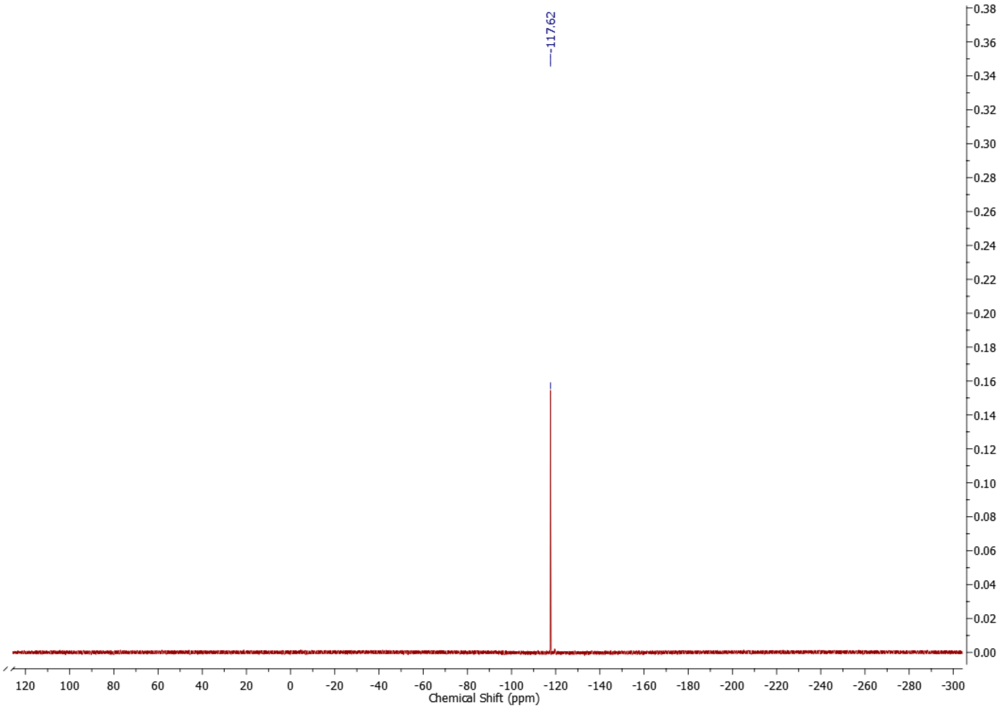

Figure S15. ^{19}F NMR spectrum of compound **4c** (471 MHz).

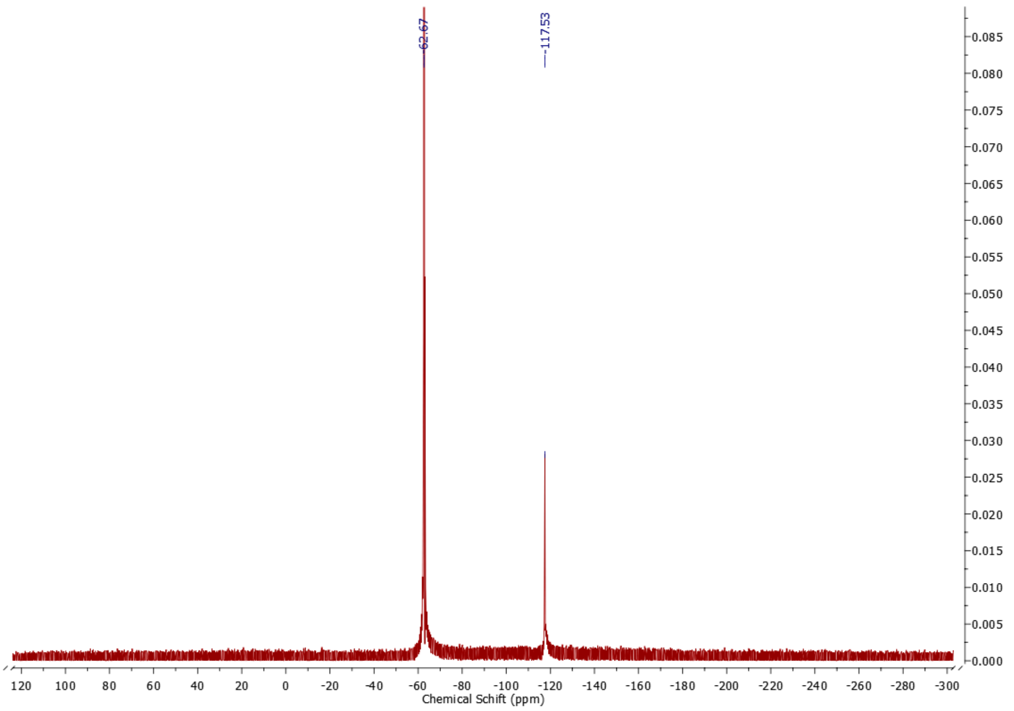

Figure S16. ^{19}F NMR spectrum of compound **4d** (471 MHz).

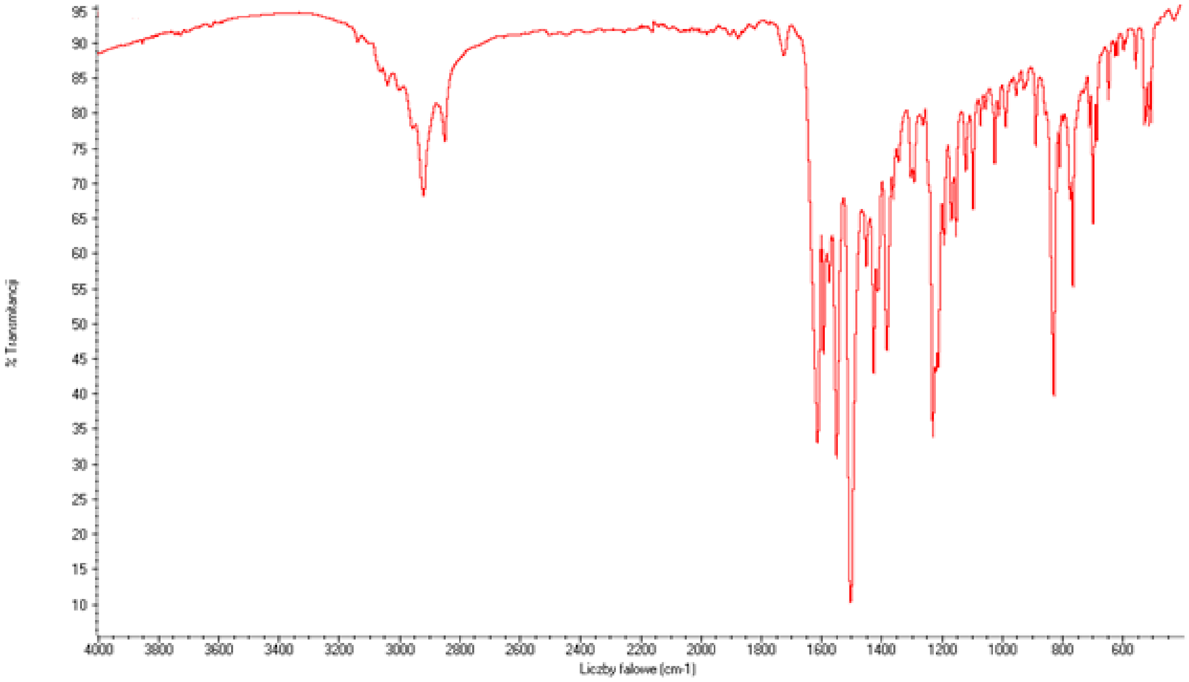

Figure S17. IR spectrum of compound **4a**.

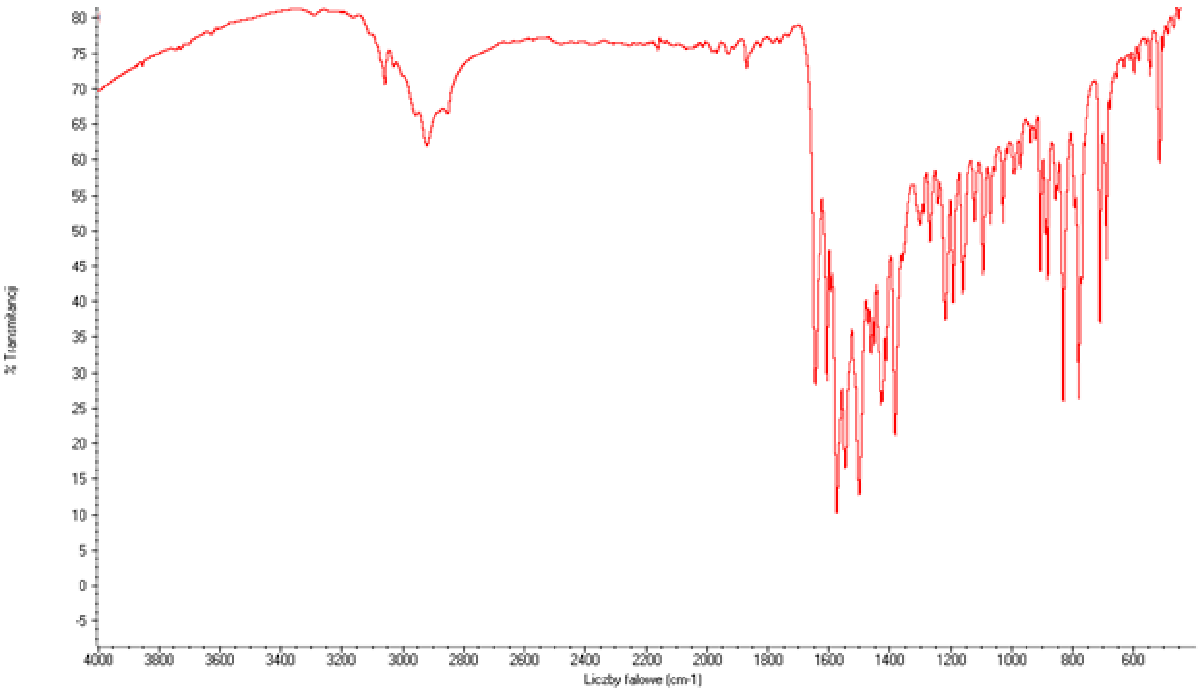

Figure S18. IR spectrum of compound **4b**.

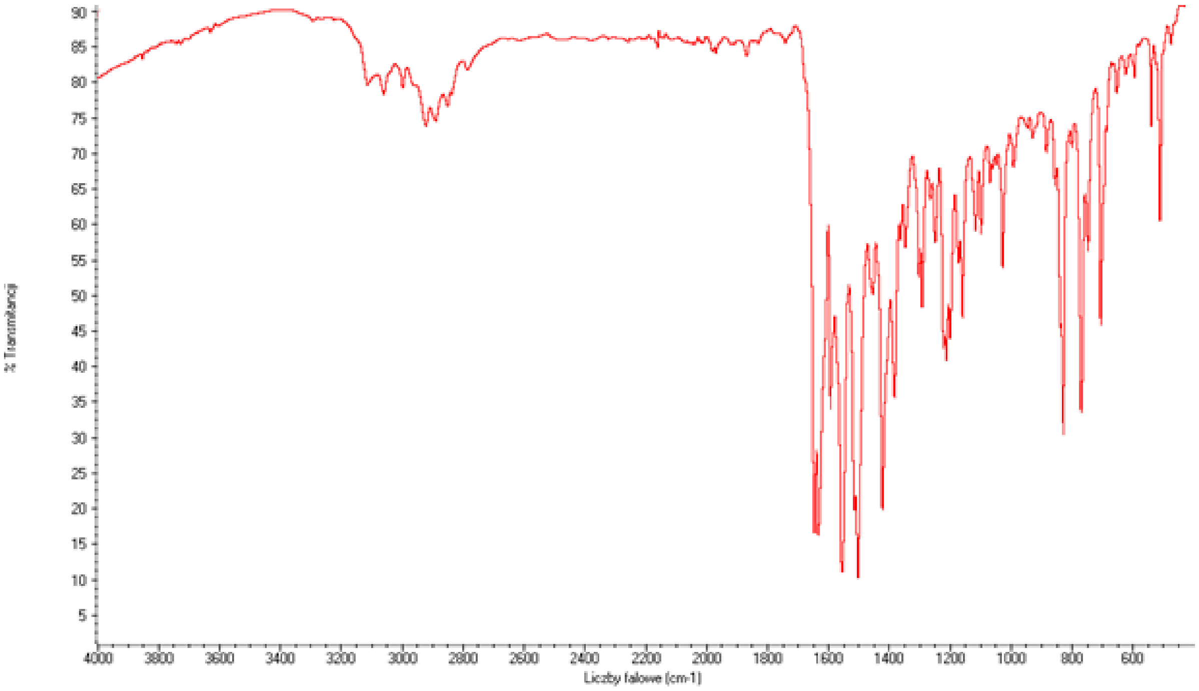

Figure S19. IR spectrum of compound **4c**.

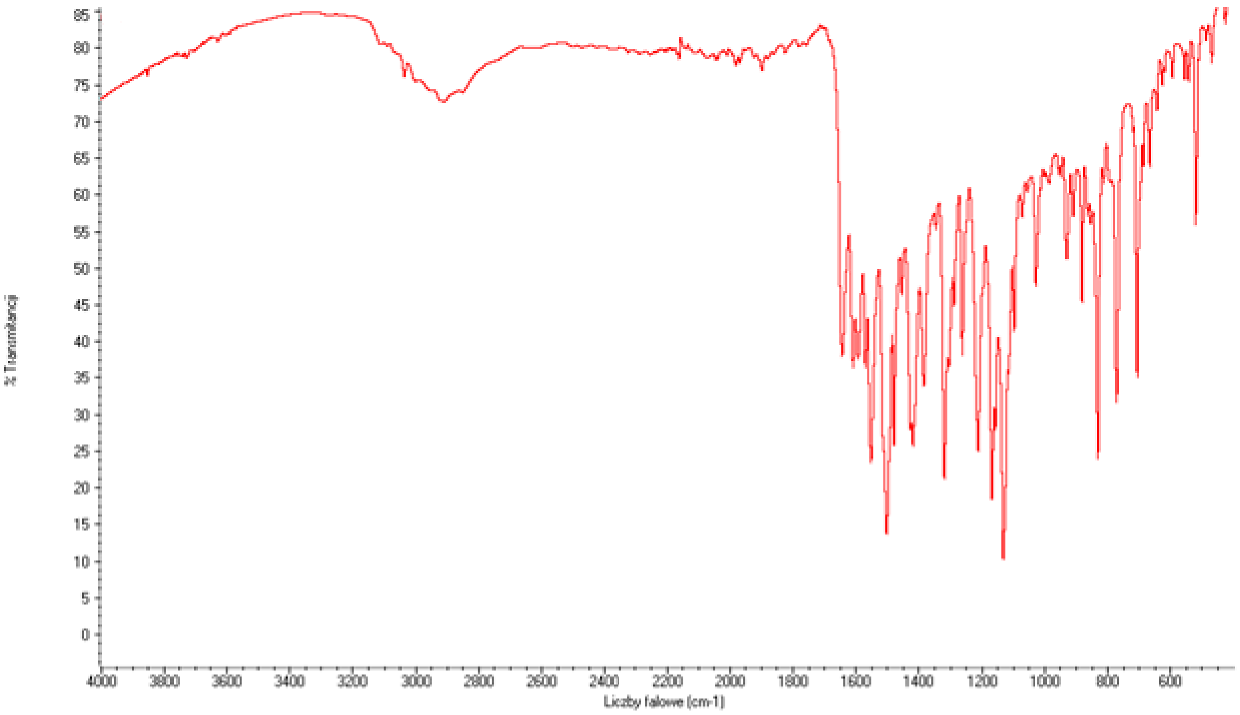

Figure S20. IR spectrum of compound **4d**.
